# Urinary Extracellular Vesicles for Renal Tubular Transporters Expression in Patients With Gitelman Syndrome

**DOI:** 10.3389/fmed.2021.679171

**Published:** 2021-06-09

**Authors:** Chih-Chien Sung, Min-Hsiu Chen, Yi-Chang Lin, Yu-Chun Lin, Yi-Jia Lin, Sung-Sen Yang, Shih-Hua Lin

**Affiliations:** ^1^Division of Nephrology, Department of Medicine, National Defense Medical Center, Tri-Service General Hospital, Taipei, Taiwan; ^2^Division of Cardiovascular Surgery, Department of Surgery, National Defense Medical Center, Tri-Service General Hospital, Taipei, Taiwan; ^3^Deparment of Pathology, National Defense Medical Center, Tri-Service General Hospital, Taipei, Taiwan

**Keywords:** Gitelman syndrome, renal tubular transporters, hypokalemia, renal tubular disease, urinary extracellular vesicles (exosomes)

## Abstract

**Background:** The utility of urinary extracellular vesicles (uEVs) to faithfully represent the changes of renal tubular protein expression remains unclear. We aimed to evaluate renal tubular sodium (Na^+^) or potassium (K^+^) associated transporters expression from uEVs and kidney tissues in patients with Gitelman syndrome (GS) caused by inactivating mutations in *SLC12A3*.

**Methods:** uEVs were isolated by ultracentrifugation from 10 genetically-confirmed GS patients. Membrane transporters including Na^+^-hydrogen exchanger 3 (NHE3), Na^+^/K^+^/2Cl^−^ cotransporter (NKCC2), NaCl cotransporter (NCC), phosphorylated NCC (p-NCC), epithelial Na^+^ channel β (ENaCβ), pendrin, renal outer medullary K1 channel (ROMK), and large-conductance, voltage-activated and Ca^2+^-sensitive K^+^ channel (Maxi-K) were examined by immunoblotting of uEVs and immunofluorescence of biopsied kidney tissues. Healthy and disease (bulimic patients) controls were also enrolled.

**Results:** Characterization of uEVs was confirmed by nanoparticle tracking analysis, transmission electron microscopy, and immunoblotting. Compared with healthy controls, uEVs from GS patients showed NCC and p-NCC abundance were markedly attenuated but NHE3, ENaCβ, and pendrin abundance significantly increased. ROMK and Maxi-K abundance were also significantly accentuated. Immunofluorescence of the representative kidney tissues from GS patients also demonstrated the similar findings to uEVs. uEVs from bulimic patients showed an increased abundance of NCC and p-NCC as well as NHE3, NKCC2, ENaCβ, pendrin, ROMK and Maxi-K, akin to that in immunofluorescence of their kidney tissues.

**Conclusion:** uEVs could be a non-invasive tool to diagnose and evaluate renal tubular transporter adaptation in patients with GS and may be applied to other renal tubular diseases.

## Introduction

Gitelman syndrome (GS) is one of the most common inherited tubulopathy with a prevalence ranging from 0.25 to 4/10,000 per population. It is caused by biallelic inactivating mutations in the *SLC12A3* gene encoding thiazide-sensitive sodium-chloride cotransporter (NCC) expressed in the apical membrane of distal convoluted tubules (DCT) (1, 2). To date, more than 450 different mutations scattered throughout *SLC12A3* have been identified in GS (1, 3, 4). Clinical characteristics include renal sodium (Na^+^) wasting with secondary hyperreninemia and hyperaldosteronism, renal potassium (K^+^) wasting with chronic hypokalemia and metabolic alkalosis, and renal magnesium wasting with hypomagnesemia, but hypocalciuria (5). The defective NCC function caused by different classes of *SLC12A3* mutations leads to the reduced sodium chloride (NaCl) reabsorption in DCT with increased luminal NaCl delivery to downstream collecting ducts (CD) responsible for NaCl reabsorption via epithelial Na^+^ channel (ENaC) and K^+^ secretion via renal outer medullary K1 channel (ROMK) and large-conductance, voltage-activated and Ca^2+^-sensitive K^+^ channel (Maxi-K). Although the expression of ENaCβ, ROMK and Maxi-K in mouse GS model has been reported to be significantly increased in both immunoblotting and immunofluorescence of mouse kidney (6), the adaptive response of upstream and downstream Na^+^ and K^+^ associated transporters in response to renal Na^+^ and K^+^ wasting in GS patients remains unknown.

Urinary extracellular vesicles (uEVs) containing membrane and cytosolic proteins, mRNAs, miRNA and signaling molecules from each renal epithelial cell type may reflect the physiological state of their cells of origin (7, 8). The isolation of uEVs had the potential to shed much insight on the health status of the kidney and expression of urinary proteins (9–11). Knepper et al. has identified more than one thousand proteins including solute and water transporters, vacuolar H^+^-ATPase subunits, and disease related proteins (12). It has been also reported that the isolated uEVs had an increased NCC abundance in patients with primary aldosteronism (13, 14) and Cushing syndrome (15) as well as a rapid increase in abundance of NCC and p-NCC in healthy subjects following the mineralocorticoid administration (16). In the inherited renal tubular disorders, uEVs have been used as a non-invasive tool to detect the defect of mutated renal tubular transporter such as NCC and Na^+^/K^+^/2Cl^−^ cotransporter (NKCC2) expression in patients with GS and Bartter syndrome, respectively (17, 18). Nevertheless, uEVs for other renal Na^+^ and K^+^ associated transporters expression has not been also investigated in GS.

The aim of this study was to evaluate the changed expression of NCC, phosphorylated NCC (p-NCC), upstream DCT such as Na^+^-hydrogen exchanger 3 (NHE3), NKCC2, downstream DCT such as ENaCβ, pendrin, as well as K^+^-secreting channels such as ROMK and Maxi-K from uEVs and representative kidney tissues in patients with GS. Results to be reported indicated that a marked attenuation of NCC and p-NCC expression from uEVs could be used as a non-invasive diagnostic biomarker for GS. Both upstream NHE3 and downstream ENaCβ and pendrin from uEVs were increased in response to salt-losing and an enhanced ROMK and Maxi-K expression were associated with renal K^+^ wasting in GS patients. These findings from uEVs were similar to those obtained from renal biopsied tissues in GS patients.

## Materials and Methods

### Study Design

The study protocol was approved by the Ethics Committee on Human Studies at Tri-Service General Hospital (TSGHIRB No.2-103-05-160 and TSGHIRB No.2-105-05-062). We prospectively collected 10 genetically confirmed GS patients. Their mutations included homozygous intronic mutation (*n* = 2), compound heterozygous mutati on (*n* = 8) in the *SLC12A3* gene encoding NCC (Table 1). Five healthy controls and three bulimic patients as hypokalemic disease controls were also enrolled. The diagnosis of bulimia was based on the American Psychiatric Association's Diagnostic and Statistical Manual, Fifth Edition (19). Clinical characteristics and laboratory examination were collected and determined. Renal biopsied tissues were collected from three different GS patients with definite *SLC12A3* mutations (compound heterozygous mutation of intronic c1670-191/p.I888_H916del, p.T60M/p.R959fs, and p.T60M/splicing c.965-1G>A+c965-977gcggacatttttgt>accgaaaattttt) and one bulimic patient. All of them had long-standing, severe hypokalemia refractory to aggressive K^+^ supplementation and significant proteinuria. Control kidney tissue was obtained from normal part of kidney in one patient with renal cell carcinoma undergoing total nephrectomy.

**Table 1:** Characteristics of *SLC12A3* mutation among 10 patients with Gitelman syndrome.

**Patients**	**Genotypes**	**Nucleotide change (NM_000339.3)**	**AA change (NP_000330.3)**	**Topological localization**
1	Compound heterozygous	c.1924C>T + c.2548+253	p.R642C + Intronic	Transmembrane + C-terminal
2	Homozygous	c.1670-191C>T + c.1670-C>T	Intronic + Intronic	Transmembrane + Transmembrane
3	Compound heterozygous	c.2875_76delAG + c.2548+253	p.R959fs + Intronic	C-terminal + C-terminal
4	Compound heterozygous	c.2129C>A + c.2875-76delAG	p.S710X + p.R959fs	C-terminal + C-terminal
5	Compound heterozygous	c.488C>T+c.2660+1G>A	p.T163M + splicing	Transmembrane + C-terminal
6	Compound heterozygous	c.1000C>T+c.1326C>G	p.R334W + p.N442K	Transmembrane + Transmembrane
7	Homozygous	c.1670-191C>T + c.1670-C>T	Intronic+ Intronic	Transmembrane + Transmembrane
8	Compound heterozygous	c.2129C>A + c.2875_76delAG	p.S710X + p.R959fs	C-terminal + C-terminal
9	Compound heterozygous	c.911C>T/c.2875_76delAG	p.T304M + p.R959fs	Transmembrane + C-terminal
10	Compound heterozygous	c.2532G>A+c.805-06insTTGGCGTGGTCTCGG	p.W844X + p.T269delinsIGVVSA	C-terminal + Transmembrane

### uEVs Studies

#### Urine Collection and uEVs Isolation

Secondary morning spot urine with forty milliliters with protease inhibitors were collected for uEVs isolation by ultracentrifugation-based protocol. The urine sample was centrifuged at 17,000 × g for 10 min at 37°C. Supernatant was then ultracentrifuged at 200,000 × g for 2 h at 4°C. The pellet was resuspended in PBS or laemmli buffer with dithiothreitol.

#### Nanoparticle Tracking Analysis

Nanoparticles from isolated uEVs were analyzed using the NanoSight NS300 instrument (NanoSight Ltd, Amesbury, UK). Following published method (20), all experiments were carried out at a 1:1,000 dilution, yielding particle concentrations in the region of 1 × 10^8^ particles ml^−1^ in accordance with the manufacturer's recommendations.

#### Transmission Electron Microscopy

uEVs pellet was carefully fixed the with enough volume of 2.5% glutaraldehyde (G5882, Sigma-Aldrich) in 0.1 M sodium cacodylate, pH 7.4 and 4% paraformaldehyde mix buffer (1:1) for 1 h at 4°C and then washed with PBS. Pre-fix the sample with 1 ml of 1% Osmium tetroxide (in ddH_2_O) for 50 min at 4°C in dark. Post-fix the sample with 5% uranyl acetate (UA) blocking overnight at 4°C. Incubate for 10 min with a graded EtOH series (50, 70, 90, 95, 100%) and followed by EPON (Resin 20 ml, DDSA 7 ml, NMA 14 ml, DMP-30 0.8 ml). The uEVs samples were analyzed with a Hitachi TEM HT7700 electron microscope operated at 60 kV.

#### Immunoblotting

For immunoblotting, the loading volume of each uEVs sample was adjusted so that the loaded amount of creatinine was constant (21, 22). SDS/PAGE was carried out on an 8% polyacrylamide gel, and proteins were transferred to Immobilon®-P membranes (Millipore, Amsterdam, The Netherlands). The primary antibodies were as follows: NSE (ab254088, Abcam, Cambridge, UK), TSG101 (ab125011, Abcam, Cambridge, UK), CD9 (GTX55564, Genetex, HsinChu City), AQP2 (sc-515770, Santa Cruz Biotechnology, Santa Cruz, CA), NHE3 (NHE31-A, Alpha Diagnostic Intl Inc., San Antonio, TX) (6), NKCC2 (AB2281, Millipore, Temecula, CA), NCC (AB3553, Millipore, Temecula, CA) (23), ENaCβ (ASC-019, Alomone labs, Jerusalem, Israel) (23), p-NCC (17T, in-house antibody) (23), Maxi-K (APC-021, Alomone labs, Jerusalem, Israel) (6), ROMK (APC-001, Alomone labs, Jerusalem, Israel) (6), and pendrin (ARP41739_P050, Aviva system biology, San Diego, CA). The membranes were incubated with the secondary antibody. Immunoreactive proteins were detected by the enhanced chemiluminescence method (Pierce, Rockford, IL, USA). The immunopositive bands from immunoblotting were quantified using pixel density scanning and calculated using Image J and the relative band intensity was normalized to the healthy controls.

### Immunofluorescence of Kidney Tissue

After paraffin removal and rehydration, the slides were heated in 1× citrate buffer (ThermoFisher) and exposed to 3% H_2_O_2_ (ThermoFisher) at room temperature and then the blocking solution. After washing with PBS plus 0.1% Tween 20 (J.T. Baker), the tissue was incubated with primary antibodies at 4°C overnight. The primary antibodies of AQP2, NHE3, NKCC2, NCC, p-NCC, ENaCβ, Maxi-K, ROMK, and pendrin were used. The tissues were exposed to species-specific secondary antibodies conjugated to Alexa Fluor fluorophores (ThermoFisher). Immunofluorescence images were obtained by Zeiss LSM880 confocal microscope.

### Statistical Analyses

Serum and urine biochemistry data were expressed as mean ± standard deviation. Correlation between uEVs particles and urine creatinine were calculated by Pearson's correlation coefficient statistic in Excel. Data analyses were performed with the Prism (v5) software (GraphPad Software). Group comparisons of renal transporters from uEVs between GS patients and healthy controls were made using a two-tailed unpaired Student's *t*-test. Statistical significance was defined as *p*-values <0.05.

## Results

### Clinical Characteristics in GS

As shown in Table 2, all GS patients (Male/Female = 9/1, age 33.4 ± 7.8 years old) were normotensive with renal Na^+^ and Cl^−^ wasting and secondary hyperreninemia (plasma renin activity, PRA 28.9 ± 14.4 ng/mL/h) but normal to high plasma aldosterone concentration (PAC) (229.4 ± 69.6 pg/mL), chronic hypokalemia (K^+^, 2.34 ± 0.45 mmol/L) with higher urinary K^+^ excretion (transtubular potassium gradient, 13.46 ± 10.91), metabolic alkalosis (HCO3^−^, 28.7 ± 3.9 mmol/L), hypomagnesemia (Mg^2+^ 0.63 ± 0.07 mmol/L), and hypocalciuria (Ca^2+^/Creatinine 0.07 ± 0.06 mmol/mmol).

**Table 2:** Clinical characteristics and biochemistries in patients with Gitelman syndrome.

**Patients**		**1**	**2**	**3**	**4**	**5**	**6**	**7**	**8**	**9**	**10**	**Mean ± SD**
SBP/DBP (mmHg)		123/65	111/68	120/80	114/78	128/70	120/64	126/64	120/70	105/84	115/68	116.2 ± 7.5/69.7 ± 7.4
Serum	Reference											
BUN (mmol/L)	2.50–8.93	5.71	5.36	7.85	4.64	6.07	5.36	7.14	4.64	4.28	5.71	5.68 ± 1.12
Creatinine (μmol/L)	61.9–106.1	79.6	88.4	114.9	53 0	106.1	106.1	88.4	97.2	70.7	97.2	90.17.0 ± 18.54
Sodium (mmol/L)	136–145	135	135	138	132	140	142	138	137	134	134	137.1 ± 3.1
Potassium (mmol/L)	3.5–5.1	2.6	1.9	2.4	2.9	2.8	2.3	2.1	2.1	1.5	2.8	2.34 ± 0.45
Chloride (mmol/L)	98–107	97	100	98	94	97	99	97	98	96	96	97.2 ± 1.7
Total Calcium (mmol/L)	2.15–2.55	2.33	2.20	2.33	2.35	2.53	2.45	2.50	2.23	2.45	2.45	2.38 ± 0.11
Magnesium (mmol/L)	0.7–1.05	0.53	0.66	0.62	0.62	0.70	0.66	0.74	0.58	0.62	0.53	0.63 ± 0.67
Hematocrit (%)	38.0–47.0	45.7	49.0	46.3	39.9	53.9	54.0	48.3	46.8	44.4	45.6	47.4 ± 4.3
Albumin (g/L)	35–57	43	37	43	48	47	46	43	38	46	45	44 ± 4
PRA (ng/ml/hr)	1.31–3.95	17.15	10.29	47.13	6.32	50.00	31.97	38.35	29.04	30.35	29.14	28.9 ± 14.4
PAC (pg/ml)	70–350	252	206	140	147	134	266	304	320	288	237	229.4 ± 69.6
HCO3^−^ (mmol/L)	24	31.6	27.2	30.7	28.0	33.0	26.8	28.0	22.6	24.1	35.1	28.7 ± 3.9
**Urine**												
Creatinine (mmol/L)		10.6	9.4	7.9	5.7	7.3	2.1	7.9	6.2	4.7	5.2	8.6 ± 3.8
Sodium (mmol/L)		172	53	58	66	64	46	96	32	42	199	82.8 ± 57.1
Potassium (mmol/L)		45	21	27	48	43	26	17	56	37	49	36.9 ± 13.3
Chloride (mmol/L)		143	86	93	67	44	59	51	35	77	207	86.2 ± 52.4
Calcium (mmol/L)		0.58	0.55	0.25	1.28	0.23	0.05	0.10	0.63	0.38	0.53	0.46 ± 0.35
Magnesium (mmol/L)		3.09	2.55	2.34	3.58	3.17	0.86	0.62	2.26	1.52	4.61	2.46 ± 1.23
TTKG		7.15	7.28	7.10	10.80	12.51	12.61	7.13	41.13	22.67	6.23	13.46 ± 10.91

*SBP, systolic blood pressure; DBP, diastolic blood pressure; PRA, plasma renin activity; PAC, plasma aldosterone concentration; TTKG, transtubular potassium gradient*.

### Characterization of uEVs

**Figure 1:**
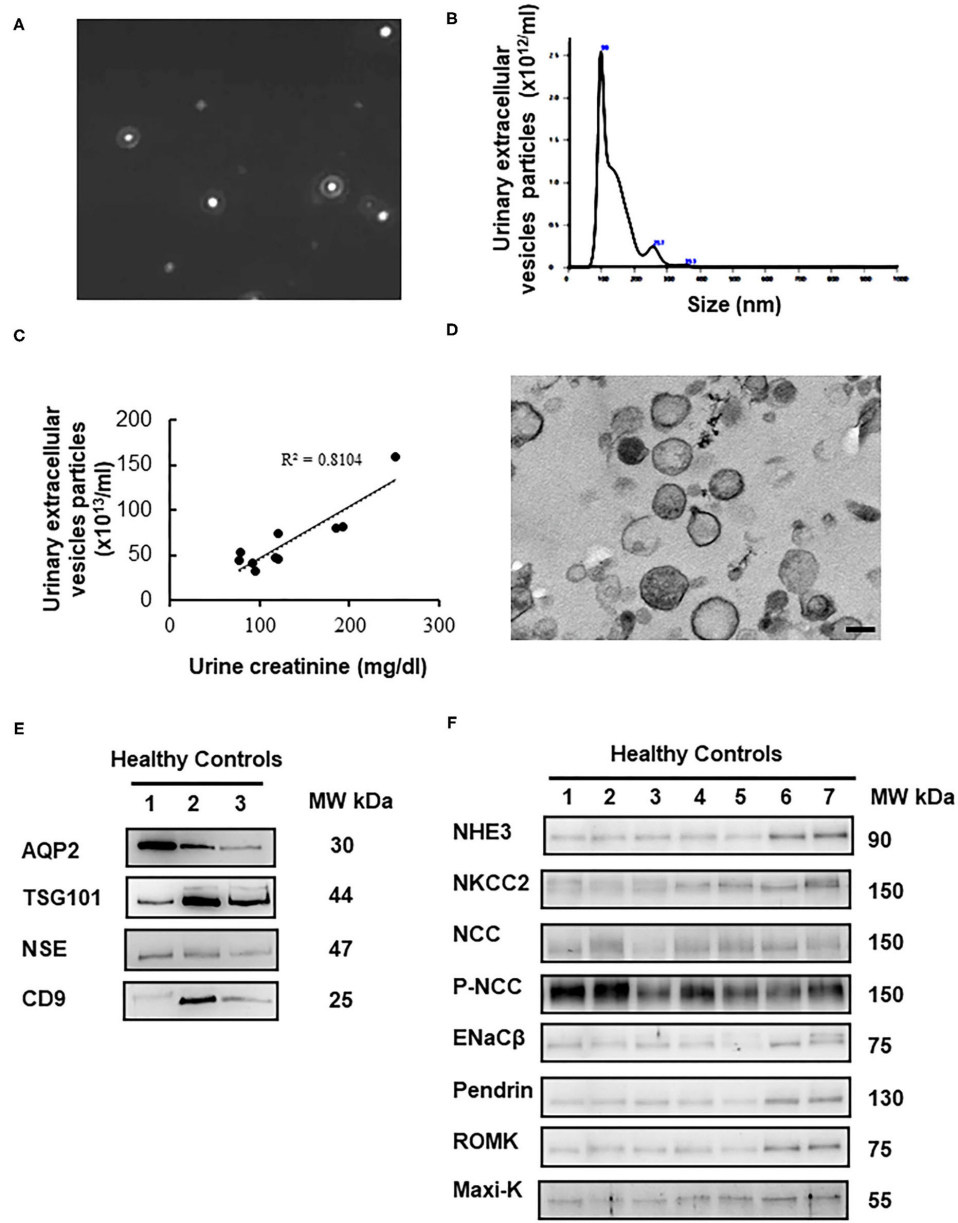
Characterization of urinary extracellular vesicles (uEVs) from healthy controls. **(A)** Screen shot from 1:2,000 diluted urine sample reveals a range of particle sizes by nanoparticle tracking analysis (NTA). **(B)** Concentration and size distribution of uEVs (0–150 nm diameter) by NTA were shown. The concentration is expressed as number of particles per ml. **(C)** uEVs particles were correlated strongly with urine creatinine (*r*^2^ for 0.81, *P* < 0.0001). **(D)** Transmission electron microscopy of uEVs was shown (scale bar 100 nm). **(E)** uEVs markers (AQP2, TSG101, NSE, and CD9) were assessed by immunoblotting. **(F)** Expression pattern of renal transporters including NHE3, NKCC2, NCC, p-NCC, ENaCβ, pendrin, ROMK, and Maxi-K from healthy controls was similar.

Characterization of the uEVs in healthy controls was validated by nanoparticle tracking analysis (NTA), transmission electron microscopy (TEM), and immunoblotting of uEVs makers. NTA identified size distribution of particles in the expected uEVs size range of 20–120 nm shown in Figures 1A,B. The mean particle size and concentration were 132.9 ± 65.8 nm and 6.6 × 10^14^/ml, respectively. uEVs number was correlated strongly with urine creatinine (*r*^2^ for 0.81, *P* < 0.0001) shown in Figure 1C. TEM also confirmed the quality of uEVs isolated by ultracentrifugation (Figure 1D). To further validate the uEVs purification protocol, we evaluated four commonly used uEVs makers including AQP2, TSG101, NSE, and CD9 in immunoblotting shown in Figure 1E. Expression pattern of selected renal transporters including NHE3, NKCC2, NCC, p-NCC, ENaCβ, pendrin, ROMK, and Maxi-K in healthy controls were shown in Figure 1F.

### uEVs for Renal Tubular Na^+^ and K^+^ Associated Transporter Expression in GS

**Figure 2:**
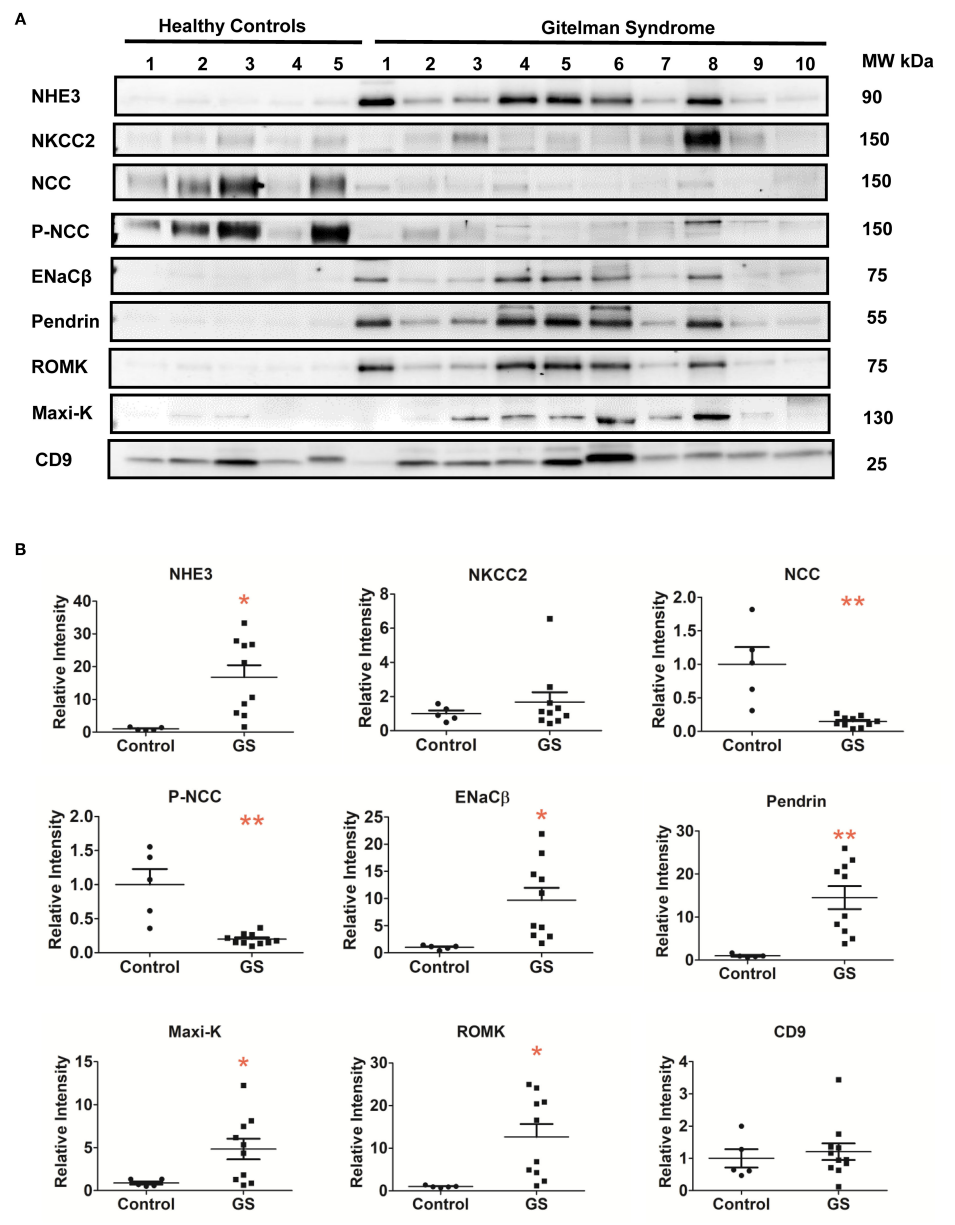
Renal Na^+^ and K^+^ associated transporters expression from urinary extracellular vesicles in patients with GS (*n* = 10) compared with healthy controls. **(A)** Immunoblotting of renal transporters (NHE3, NKCC2, NCC, p-NCC, ENaCβ, pendrin, ROMK, Maxi-K, and CD9). **(B)** Quantification of immunoblotting of NHE3, NKCC2, NCC, p-NCC, ENaCβ, pendrin, ROMK, Maxi-K, and CD9. Error bars, standard deviation. *P < 0.05, **P < 0.01.

Compared with healthy controls, GS patients with different biallelic mutations exhibited a markedly attenuated expression of NCC and p-NCC protein isolated from their uEVs, indicative of an impaired NCC expression and function in GS (Figures 2A,B). The expression of NHE3, ENaCβ, and pendrin significantly increased although NKCC2 was not significantly increased. For uEVs associated renal tubular K^+^ associated transporter expression, GS patients had significantly increased ROMK and Maxi-K expression.

### Renal Tubular Na^+^ and K^+^ Associated Transporter Expression From Kidney Tissues in GS

**Figure 3:**
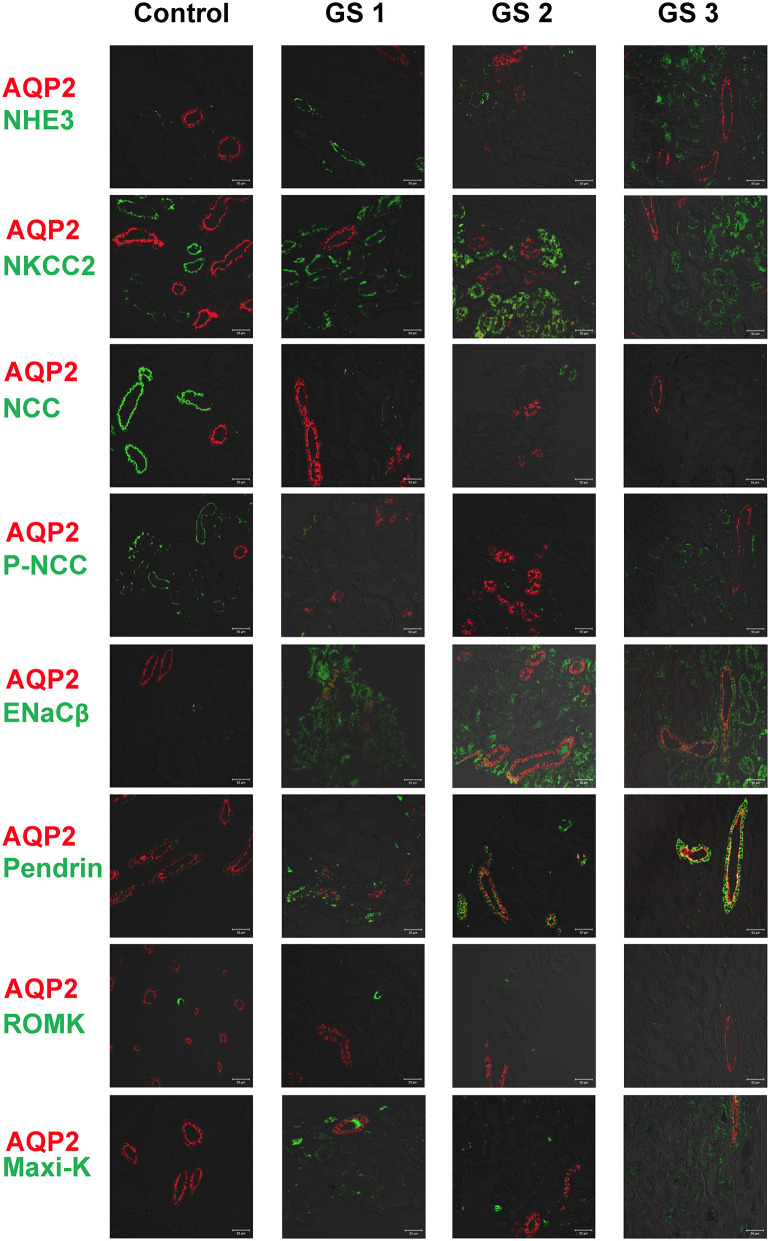
Immunofluorescence of biopsied kidney tissues from another 3 representative GS patients (GS 1, GS 2, and GS 3) compared with the control kidney tissue. Renal transporters including NHE3, NKCC2, NCC, p-NCC, ENaCβ, pendrin, ROMK, Maxi-K were stained with green. AQP2 was stained with red for localization. Scale bar, 50 μm.

AQP2 used for a tubular maker of CD was clearly stained. Compared with control kidney tissue, the representative kidney tissues from GS patients showed obviously diminished expression in both NCC and p-NCC. The expression of NHE3, ENaCβ and pendrin was significantly increased (Figure 3). The expression of ROMK was increased and the Maxi-K unexpressed in control kidney tissue without hypokalemia was also significantly enhanced in three GS patients. Overall, these finding from immunofluorescence of kidney tissues supported the findings of the isolated uEVs to examine Na^+^ and K^+^ associated renal transporter adaptation in GS patients.

### Tubular Transporter Expression From uEVs and Kidney Tissue in Bulimic Patients

**Figure 4:**
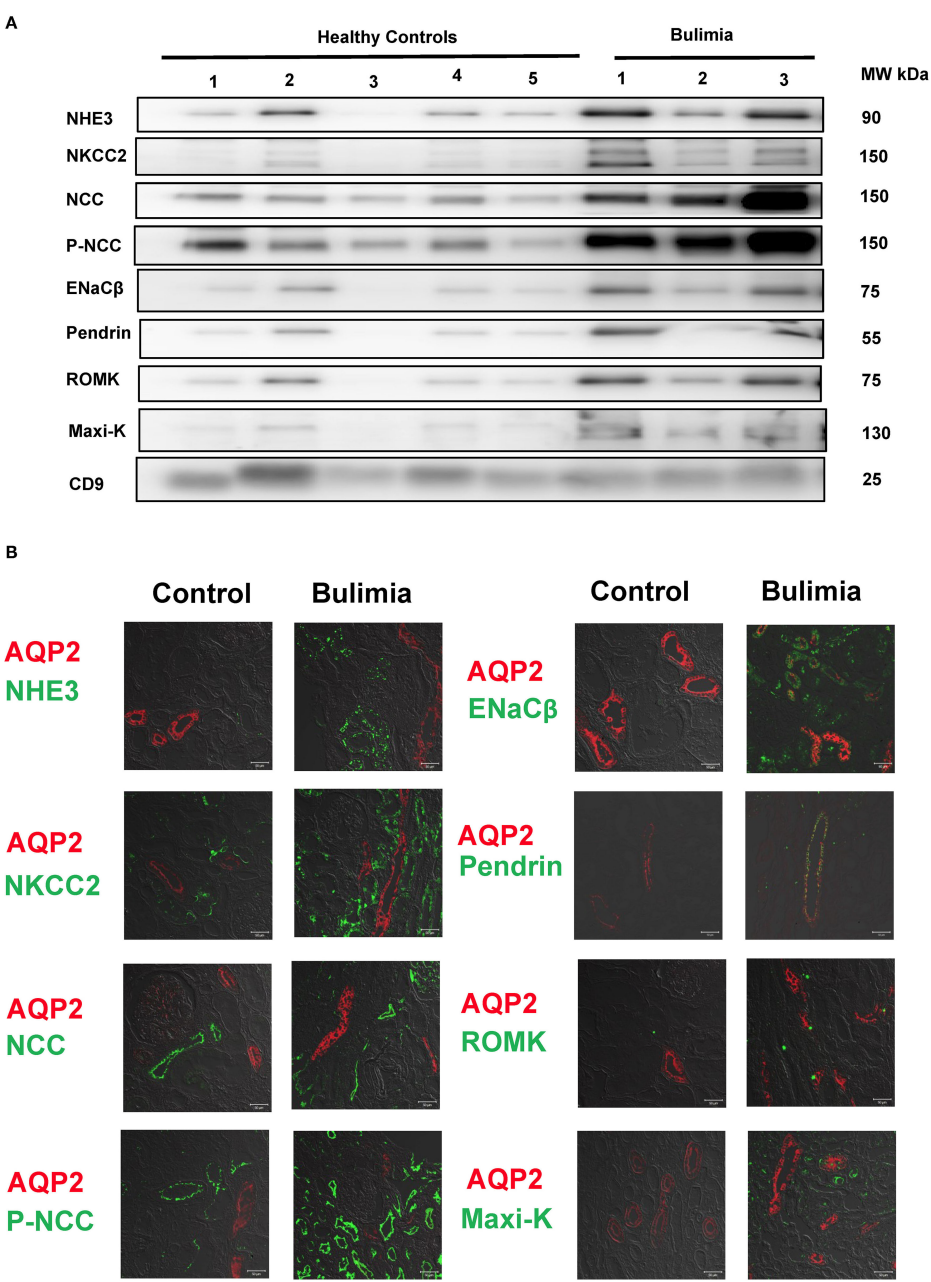
Renal transporters expression from urinary extracellular vesicles (uEVs) and immunofluorescence of biopsied kidney tissues from bulimic patients. **(A)** Immunoblotting of renal transporters (NHE3, NKCC2, NCC, p-NCC, ENaCβ, pendrin, ROMK, Maxi-K, and CD9) from uEVs in bulimic patients (*n* = 3) compared with healthy control. **(B)** Immunofluorescence of NHE3, NKCC2, NCC, p-NCC (green, **right**) and ENaCβ, pendrin, ROMK, Maxi-K (green, **left**) from one representative bulimia patient compared with the control. AQP2 was stained with red for localization. Scale bar, 50 μm.

Three bulimic patients (male/female = 2/1, age 23.3 ± 4.0 years old) with normotension (systolic blood pressure 102 ± 17 mmHg, diastolic blood pressure 63 ± 5 mmHg) exhibited chronic hypokalemia (K^+^ 2.73 ± 0.55 mmol/L), metabolic alkalosis (HCO3^−^, 46.6 ± 11.9 mmol/L), with secondary hyperreninemia (PRA 4.3 ± 1.3 ng/mL/h) but normal to high PAC (127.6 ± 26.7 pg/mL). They all exhibited higher urinary K^+^ excretion, high Na^+^ (120.3 ± 80.4 mmol/L) but low Cl^−^ (18.7 ± 6.4 mmol/L), alkaline urine (bicarbonaturia), indicative of recent vomiting. As shown in Figure 4A, uEVs from them showed an increased abundance of NCC and p-NCC as well as NHE3, NKCC2, ENaCβ, pendrin, ROMK and Maxi-K. Immunofluorescence of the kidney tissue from a representative bulimic patient also had the similar finding to those in uEVs (Figure 4B).

## Discussion

In this study, the isolated uEVs from GS patients with biallelic *SLC12A3* mutations showed the markedly attenuated expression of NCC and p-NCC whereas those from non-GS bulimic patients did a significantly enhanced abundance of NCC and p-NCC. In response to renal salt loss, the expression of upstream NHE3 and downstream ENaCβ, and pendrin were all accentuated. The abundance of ROMK and Maxi-K expression were also augmented for renal K^+^ wasting in GS. Immunofluorescence of the representative kidney tissues from GS and bulimic patients also demonstrated similar findings to those from uEVs. This study might be the first to assess the abundance of renal tubular Na^+^ and K^+^ associated transporters from uEVs and kidney tissues in GS patients.

GS caused by inactivating *SLC12A3* mutations has an impaired NCC expression and/or activity as shown in both *vitro* and *vivo* studies. Although normal NCC expression with an impaired functional activity was shown in oocytes overexpressed T60M mutation at the critical NCC phosphorylation site, a markedly decreased total NCC and p-NCC protein abundance was evident in NccT58M/T58M GS knock-in mice and in the urine of human GS with homozygous T60M mutations (23). In addition, the reduced or abolished NCC abundance on the apical membrane of DCT from the human kidney tissues in GS patients with *SLC12A3* mutations were also demonstrated (24, 25). These findings supported the notion that the reduced expression of NCC was a biomarker for GS despite different mechanisms involved in the impaired NCC protein synthesis (24, 26), and sorting or trafficking defect of NCC (27). Accordingly, it is important to find a non-invasive method to faithfully represent NCC abundance in GS. Previous studies using uEVs to measure the mutated NCC by immunoblotting and enzyme-linked immunosorbent assays (ELISAs) in GS patients only showed the decreased NCC abundance (17, 18). In this study, the isolated uEVs from GS patients revealed that both NCC and p-NCC abundance were markedly diminished, also confirmed by the human kidney tissue of genetically-confirmed GS patients.

It is of great interest to understand and localize the tubular adaptation in the inherited renal tubular disorders. The traditional methods were the preparation of whole kidney sections for immunostaining and immunoblotting or biotinylating the rat or mice kidney tissues *in situ* under various chronic conditions in animal models. Tubular adaptation to renal Na^+^ loss has been evaluated in the distal tubules in experimental models of GS but not human GS. Knepper et al. has used the LC-MS/MS to profile the proteome of human uEVs and suggested that uEVs analysis be a potential approach to discover adaptation in renal transporters (12). Using uEVs analysis in GS, we found that the abundance of upstream NHE3 in the proximal tubules (PT) necessary for bicarbonate reabsorption, salt and fluid homeostasis was significantly increased (28–30). Renal NHE3 abundance was markedly increased in K^+^-depleted rats (31), indicating that NHE3 expression can be also regulated by the hypokalemia independent of volume depletion. Similarly, downstream ENaCβ in the principal cells of CD for tubular salt reabsorption was enhanced (32, 33). Of note, pendrin as a Cl^−^/HCO3^−^ exchanger expressed in the apical region of distal tubules and involved in the tubular Cl^−^ absorption and HCO3^−^ secretion was augmented (34). Activation of pendrin-mediated Cl^−^ absorption has also been reported in NCC KO mice (35). Although pendrin expression has been examined in many rodent treatment models such as NCC KO mice, an aldosterone infusion or the administration of NaHCO_3_ to regulate acid-base and salt regulation, our study suggested the increased pendrin expression from uEVs and biopsied kidney tissues be responsive to renal salt wasting and also chronic metabolic alkalosis in GS patients.

K^+^ excretion in distal nephron is driven by either voltage-dependent ROMK and/or flow dependent Maxi-K (36). ROMK is an inwardly rectifying K^+^ channel (37) traditionally responsible for the main renal K^+^ secretory channel, dependent on Na^+^ delivery and driven by electrogenic ENaC-mediated Na^+^ reabsorption (38–40). Maxi-K is flow-stimulated K^+^ secretion and activated by an increase in intracellular calcium and membrane depolarization (41, 42). Defective NaCl absorption in DCT leads to the increased flow rate to downstream connecting tubules (CNT) and CD to naturally stimulate both ROMK and Maxi-K. In animal model of GS (Ser707X knockin mice), an enhanced expression of both ROMK and Maxi-K has been clearly shown (6). Our uEVs for the expression of both ROMK and Maxi-K abundance were significantly increased in GS patients, akin to the findings of their representative immunofluorescence of kidney tissues. Of note, the abundance of Maxi-K was extremely low in both uEVs and biopsied kidney in controls but higher in GS patients, indicating that Maxi-K expression was more augmented at the high urinary flow rate.

The above-mentioned findings with an increased protein expression related to Na^+^ reabsorption, K^+^ secretion and regulation of acid/base balance at distal nephron from the uEVs in our GS patients with diminished NCC expression consisted with current idea that distal tubules including CD are highly plastic. Tubular plasticity for adaptation is defined as structural remodeling of renal tubules via cell proliferation (hyperplasia) and cell growth (hypertrophy) (43, 44). In NCC-deficient mice, early DCT showed a remarkable atrophy but CNT exhibited a marked epithelial hypertrophy accompanied by an increased apical abundance of ENaC (45). In SPAK KO mice featuring GS-like phenotypes, a distal nephron remodeling process of the CNT/CD developed to produce an increase in the numbers of principle cells and β-intercalated cells (46). These two mice models with deficient NCC clearly demonstrated the markedly attenuated DCT along with the distinctly hypertrophic and/or hyperplastic CNT/CD. Our uEVs results in GS patients were similar to those from NCC deficient animal studies, also supporting the notion of nephron plasticity with compensatory increase in the CNT/CD size.

Bulimic patients, also called pseudo-GS syndrome (47, 48), exhibiting similar laboratory and clinical features to GS, were also evaluated for disease controls. In contrast to GS patients, uEVs from bulimic patients showed a markedly enhanced abundance of NCC and p-NCC. The increased NCC and p-NCC abundance may be secondary response to volume depletion and K^+^ deficiency *per se*. In rat model of K^+^ deficiency, enhanced abundance NCC and p-NCC has been clearly shown (49), closely linked to increased WNK body formation and activation of SPAK/OSR1 (50). Similarly, uEVs for upstream NHE3 and NKCC2 along with downstream ENaCβ and pendrin expression were also increased in response to salt-losing and metabolic alkalosis. Of interest, only the slightly increased ROMK and Maxi-K abundance from the isolated uEVs and biopsied kidney tissues may be associated with the interaction of bicarbonaturia to stimulate them as well as the enhanced NCC and chronic hypokalemia to suppress them.

Recently, uEVs has been emerged as a promising liquid biopsy biomarker in kidney disease research. Several novel biomarkers from uEVs including proteins, miRNA or non-coding RNA have been discovered in acute kidney injury (51, 52), chronic kidney disease (53, 54), diabetic nephropathy (55), focal segmental glomerulosclerosis (56), and lupus nephritis (57). In addition to GS and Bartter syndrome, uEVs is also utilized in some renal tubular disorders such as nephrogenic diabetes insipidus, and familial hyperkalemic hypertension due to *KLHL3* mutation (58). Accordingly, these evidence demonstrated the relevance of uEVs in understanding the pathophysiology of kidney diseases and the discovery of potential therapeutic targets. Our study provided a feasible way to analyze the differential expression proteins in renal tubular disorders and may be also applied to other non-tubular disorder such as cisplatin or drug induced tubulopathy.

There were some limitations of this study. First, the sample size of GS patients was still small due to the restricted loading wells of SDS/PAGE for immunoblotting. Second, other relevant transporters along the renal tubules such as TRPV5 and TRPM6 were not examined because of limited uEVs proteins isolated from ultracentrifugation. Third, the localization of these transporters in renal tubules could not be identified using uEVs. Finally, the specificity and sensitivity of the antibodies used for this study might affect expression of renal transporters between immunoblotting and immunofluorescence (for example NKCC2). Using the detergent for immunoblotting is another approach to enhance intracellular epitope recognition in uEVs (22).

In conclusion, uEVs could be used as non-invasive diagnostic tool to evaluate the renal tubular Na^+^ or K^+^ associated transporters expression in GS patients. High-throughput proteomic studies from uEVs in GS patients will be anticipated in the further investigation.

## Data Availability Statement

The original contributions presented in the study are included in the article/supplementary material, further inquiries can be directed to the corresponding author/s.

## Ethics Statement

The studies involving human participants were reviewed and approved by Institutional Review Board of the Tri-Service General Hospital of Taiwan (TSGHIRB No.2-103-05-160 and TSGHIRB No.2-105-05-062). The patients/participants provided their written informed consent to participate in this study.

## Author Contributions

C-CS, M-HC, and S-HL substantially contributed to study conception and design, acquisition of data, and analysis and interpretation of data. Y-ChaL, Y-ChuL, Y-JL, and S-SY substantially contributed to acquisition of data, and analysis and interpretation of data. All the authors revised the paper and approved the final version of the article to be published.

## Conflict of Interest

The authors declare that the research was conducted in the absence of any commercial or financial relationships that could be construed as a potential conflict of interest.
